# The Ability to Manage Unexpected Events and the Vocational Identity in Young People: The Italian Validation of Planned Happenstance Career Inventory

**DOI:** 10.3389/fpsyg.2022.899411

**Published:** 2022-06-30

**Authors:** Luigia Simona Sica, Michela Ponticorvo, Tiziana Di Palma

**Affiliations:** Department of Humanistic Studies, University of Naples Federico II, Naples, Italy

**Keywords:** planned happenstance, vocational identity, young people, Italian validation, career inventory

## Abstract

The present study had two goals: to test the validity of Planned Happenstance Career Inventory (PHCI) in the Italian context and to explore the relations between PHC skills and vocational identity processes within a sample of 472 undergraduate students attending university in the southern of Italy. Moreover, we examined relations between the PHCI and measures of vocational identity processes. With regard to the first goal, results show that for the Italian version of the instrument was confirmed the multifactor structure of the original version. The multi-group analyses showed that invariance between genders is supported. Convergent validity and divergent validity of the measure were reported. Concerning the second goal, the skills of planned happenstance show different associations with Vocational Identity Dimensions. As hypothesized, the exploration and commitment dimensions of vocational identity status are positive related to Planned Happenstance Skills. These findings suggest implications for career guidance and counseling.

## Introduction

The current world of work represents an unpredictable territory for young people, characterized by constant changes and numerous contractual forms that make school to work/university transition a difficult moment for the formation of professional identity. Furthermore, the recent pandemic situation (Staguhn et al., 2021) has made more necessary to cope with unforeseen events and flexibility in defining one’s vocational identity. Therefore, as of right now, even more crucial it is the identification of both resources and strategies to help young people to manage and use unexpected events constructively.

Recent research has emphasized the importance of identifying psychological resources suitable for facing the challenges posed by the need to make a choice in the vocational domain (Sica and Aleni Sestito, 2021). In the present study, we intend to broaden this resource identification, focusing on the study of a dimension that is strictly connected to the unpredictability and flexibility of the world of youth work, the planned happenstance (Mitchell et al., 1999), i.e., the ability to use unexpected events as a possibility of development (Kim et al., 2014). This could be an important resource for vocational identity for both support for the vocational identity commitment making process and protective factor against identity distress. According to this assumption, planned happenstance could be a resource to cope with unpredictable experiences (e.g., pandemic).

In order to accomplish this, it is also relevant to identify measurements that are usable in specific context in an effective way. Thus, in the present study, we examined the validity of the Italian version of the Planned Happenstance Career Inventory (PHCI; Kim et al., 2014), a scale for assessing skills related to capitalizing on opportunities for career development and decision-making. We also examined relation between the PHCI and measures of vocational identity processes.

## The Importance of Work in the Identity Formation of Late-Adolescence in Italy

Building a vocational identity and a professional definition of self is a major task in adolescence (Erickson, 1968; Savickas, 1985; Porfeli et al., 2011). Vocational identity is conceived as a domain-specific aspect of overall identity, providing young people with a framework to regulate the pursuit of their academic and career objectives (Hirschi, 2012). People exhibiting an advanced identity status show greater career planning and decidedness (Wallace-Broscious et al., 1994) and a more advanced identity (Kroger, 1988; Meeus, 1996; Skorikov and Vondracek, 1998). Vocational identity is, therefore, believed to be a defining feature in adolescent and young adult life, a leading aspect of global identity development.

The close relation between identity and vocational identity was first assumed by Erickson (1968) and this has been reported by several studies (Savickas, 1985; Skorikov and Vondracek, 1998; Nauta and Khan, 2007; Aleni Sestito et al., 2015). Although vocational identity development is shown to be stable on a population level while transitioning to adulthood, some degree of variability is observed in the pathways and timing of vocational identity progress from both individual and contextual perspectives (Aleni Sestito et al., 2015). Specifically, emerging adults in the modern era may experience a prolonged period of identity moratorium as they explore major life roles in the absence of making solid commitments (Crocetti and Palmonari, 2011). This experience may be particularly amplified for college students who tend to delay these transitions more so than those who do not pursue higher education (Crocetti et al., 2012). Moreover, college students may show a greater diversity of identity statuses revolving around identity diffusion, disengagement, and indifference (Sica et al., 2014; Di Palma and Reid, 2019) and these statuses may be adaptive within political-economic conditions, like those in Italy, limiting job opportunities and viable career pathways.

Thus, the impact of global economic changes on the nature of work and career and how that may relate to changes in vocational identity processes and structure (Ashkenas et al., 2015) serves as a backdrop for the present study. Opportunities in the modern labor market include a proliferation of new careers and contracts, and the flexibility to redefine one’s self and career in a way that is becoming increasingly accepted and, in some cases, normative and even admired (Arthur and Rousseau, 1996). In sum, the global economy presents challenges to identity and career in terms of job security and predictability.

Nevertheless, as highlighted by many career scholars (Arthur and Rousseau, 1996; Hall, 2004), since accountability for career development belongs now to the individual, it becomes more and more important for personal choices to create a personal path that leads one’s own career, and thus fundamental for employability (Fugate et al., 2004). Hence, the need to identify and measure psychological skills that can be supported and strengthened in order to help young people define their own identity and vocation.

One part of the literature related to career counseling (Van Vianen et al., 2009; Savickas and Porfeli, 2012) has focused on self-regulatory resources denoted as concern, control, curiosity, and confidence (career adaptability resources). These have been conceptualized as self-regulation strengths that a person may draw upon to solve unfamiliar and complex problems presented by developmental tasks, occupational transitions, and work traumas. The other part focuses on the specific ability to transform unexpected events and uncertainty into a real resource; the planned happenstance theory. We refer to the latter in this study since it explores the ability to transform uncertainty into possibility for individual learning and development. According to us, this constitutes a step forward, going beyond the ability to control and cope with moments of transition and change. So, it seems to us that this could be a key factor in managing of unexpected events which are related not only to personal biography but also refers to a greater malleability of the context in the current era. Furthermore, it seems to us more congruent with the management of unexpected events which are, not only related only to the personal autobiography but also refer to a greater malleability of the context in the current era.

## Managing Uncertainty: The Planned Happenstance Theory and Measurement

Planned happenstance theory derived from the learning theory of career counseling (Krumboltz, 1996), which was an expansion of the social learning theory of career decision-making (Krumboltz, 1979). It was proposed by Mitchell et al. (1999) as conceptual framework extending career counseling to include the creating and transforming of unplanned events into opportunities for learning. In applicative terms, the planned happenstance theory could support people to generate, recognize, and incorporate chance events into their career development. As proposed by Mitchell et al. (1999), planned happenstance theory includes two main assumptions: exploration generates opportunities to improve quality of life, and skills enable people to seize opportunities. Mainly, planned happenstance theory identified five skills to recognize, create, and use chance as career opportunities: Curiosity (exploring new learning opportunities); Persistence (exerting effort despite setbacks); Flexibility (changing attitudes and circumstances); Optimism (viewing new opportunities as possible and attainable); and Risk-Taking (taking action in the face of uncertain outcomes).

The planned happenstance was also studied in relation to vocational identity statuses (Rhee et al., 2016). Specifically, more advanced vocational identity statuses (achieved and searching moratorium) were found to have higher scores in the assessment of planned happenstance skills than their counterpart, the less advanced group (diffused and undifferentiated). Yang et al. (2017) explored happenstance dimensions’ changes during the transition from school to work. They found that among the five PH skills, curiosity, flexibility, persistence, and optimism significantly declined, while risk-taking did not show significant change over time. They also found that the trajectories of curiosity and persistence varied by the degree of career aspiration and career barriers. Valickas et al. (2019) explored the role of planned happenstance skills when predicting psychological well-being and academic adjustment, highlighting that planned happenstance skills were a significant predictor of academic achievement and psychological well-being.

### Scales for Planned Happenstance

According to the planned happenstance theory (Mitchell et al., 1999), Kim (2012, Unpublished)^1^ developed the Career-Related Planned Happenstance Scale (CPHS). The CPHS consists of 14 items, written in Korean, measuring the five skills (curiosity, persistence, flexibility, optimism, and risk-taking) that can create and transform unplanned events into opportunities in career-related fields.

The CPHS showed relatively low reliability (Cronbach’s *α* = 0.50) for risk-taking and flexibility.

In Kim et al. (2014) revisited the items of the CPHS and developed the Planned Happenstance Career Inventory (PHCI), the instrument that measures planned happenstance skills, to which this study refers. A total of 1.009 Korean undergraduate students (459 female, 550 male) participated in the study. Authors reported that “for the CFA, a total of 399 undergraduate students (152 female, 247 male) with a mean age of 25.35 years (SD = 1.66) participated in the study. Another sample of 185 students (99 female, 86 male) was recruited to assess construct validity by comparing the relations between the PHCI and other career-related scales. These 185 participants had a mean age of 22.10 years (SD = 2.37)” (2014, 245). After item generation and exploratory factor analysis, 130 original items were reduced to 25 items across 5 factors (5 items for each factor). A first-order factor model solution demonstrated an adequate fit to the observed data (TLI = 0.91, CFI = 0.92, RMSEA = 0.06 [0.05, 0.07], SRMR = 0.07). Multi-group confirmatory factor analysis confirmed the validity of the 5-factor structure, and the goodness of fit showed an adequate fit for both women and men models.

Lee et al. (2019) examined the cross-cultural validity of the Planned Happenstance Career Inventory (PHCI) for a sample of 262 U.S. college students. Results of this study supported the original 5-factor structure of the Korean PHCI in the U.S. sample. Authors reported that the five-factor model of the PHCI–English version yielded CFI (0.91) and NNFI (0.90) values close to 1.00, which indicated that the five-factor model had an adequate fit. Likewise, the RMSEA of 0.06 (90% confidence interval [0.05, 0.07]) indicated a fair fit, and an SRMR of 0.07 indicated a satisfactory fit” (2017, 372). Further analysis with career decision self-efficacy and vocational identity status showed evidence of convergent and divergent validity of the measure.

## The Current Study

Since 1999, Mitchell, Lewin and Krumboltz underlined the importance of flexibility and focus on change to support young people in defining their career plan, showing how these elements were not effectively recognized by career counseling. Savickas (1997) claimed for new resources to cope with the characteristics of XX century, defining as “adaptability” the capacity to manage uncertainty. Given social and career-related changes due to the pandemic, the unpredictability of life events and life conditions has become a central issue for psychologists. Starting from this assumption, a focus on skills that could enhance flexibility and ability to manage uncertainty is needed, especially during the transition from school to work, when late adolescents and young adults are facing their developmental challenges linked to the definition of their vocational identity. In this transition, the importance of a thorough and thoughtful choice, based on processes of exploration and commitment to identity, is a predictor of personal well-being and adaptation (Fusco et al., 2020). Therefore, it is important to identify indications for interventions to support the consolidation of vocational identity from a positive developmental psychology perspective that aims to support optimal development trajectories. Recent research explored resources connected to identity formation, namely creativity and agency (PSID; Sica and Aleni Sestito, 2021) and specifically to vocational identity (creativity; Sica et al., 2022); few specific attentions were paid to planned happenstance in relation with vocational identity (Rhee et al., 2016) during the transition from school to work (Yang et al., 2017; Valickas et al., 2019) and no attention was paid to the planned happenstance theory in Italy, and to its relation with well-being and/or ill-being.

Thus, according to the general hypothesis that the career planned happenstance skills could be considered as resources that do allow young people to cope with occupational concerns (vocational identity formation), this study is guided by two aims:

1.To examine the cross-cultural validity of the Planned Happenstance Career for a sample of 472 Italian late adolescents. The development of a reliable measurement of planned happenstance skills that can be used across diverse cultures has become a concern for many researchers (Kim et al., 2014; Lee et al., 2019). Therefore, according to the two previous studies on PHCI measure, we expected that the five-factor structure identified by Kim et al. (2014), and confirmed in the English version (Rhee et al., 2016; Lee et al., 2019), could emerge as appropriate for the Italian version of the measure; the solution should be consistent across genders.

2.To explore the relation between PHC skills, vocational identity processes. We expect that in the Italian context, relations similar to those reported by Rhee et al. (2016) could emerge; namely, the exploration and commitment dimensions of vocational identity status were related to higher levels of persistence and optimism, whereas the reconsideration dimension was positively related to flexibility.

## Materials and Methods

### Participants and Data Collection

Participants were 472 undergraduate students (335 women, 133 men, 4 who did not reply) enrolled at a university in southern Italy in different faculties. The sample included mainly freshmen (96.8%), 1.7 were enrolled to other years and 1.5% were unidentified. The mean age was 20.52 years (SD = 8.62). Participants were for the 55.2% commuting, 32.8% resident, 10.3% non-resident, and 1.7% did not declared their status. Most participants (98.2%) had Italian nationality, 1.2% were not of Italian nationality, and 0.6% did not declared their nationality.

The research team members who were fluent in English translated and back translated the English version of the PHCI (Lee et al., 2019). Afterward, researchers had a series of consensus meetings to review the translation and discuss the clarity and relevance of the items. The final translated version was used in the present study and is reported in Appendix 1. The study was approved by the internal university ethical committee.

The measures were administered during class time. Two researchers familiar with the survey attended classes to assist the respondents with questions about the survey. Participation in the study was voluntary and anonymity was guaranteed, and the respondents did not receive payment for their participation. Completion time was between 20 and 40 min. Of the total number of respondents, 90% took part in the research.

### Measures

#### Planned Happenstance Skills

The Italian version (Planned Happenstance Career Inventory_Italian; PHCII) of the Planned Happenstance Career Inventory (PHCI; Kim et al., 2014) was used to measure five PH skills considered crucial for recognizing, creating, and taking advantage of unexpected events as opportunities. The inventory is composed of 25 items, which are further divided into five subscales based on the Planned Happenstance theory (Mitchell et al., 1999): curiosity (e.g., “I am intrigued by the idea of an occasional opportunity leading to a whole new experience”), persistence (e.g., “I would persist in my efforts despite any unexpected barriers”), flexibility (e.g., “I am flexible about considering multiple options rather than pursuing only one career path”), optimism (e.g., “I think my future is full of possibilities”), and risk-taking (e.g., “I am ready to take risks to a certain extent in pursuing my career”). Each subscale had five items, and a 5-point Likert scale ranging from 1 (strongly disagree) to 5 (strongly agree) was used. The Italian version is reported in Appendix 1.

For the original version by Kim et al. (2014), the factor structure of the Korean version was confirmed by exploratory factor analysis and CFA. The internal consistency of the subscale scores ranged from 0.76 to 0.90, in the cited study. The convergent validity for the PHCI was demonstrated through its significant correlations with career-related scales, including career stress, career decision self-efficacy (Kim et al., 2014), and career preparation behavior (Kim and Kim, 1997).

#### Vocational Identity

The Italian version (Aleni Sestito et al., 2015) of the Vocational Identity Status Assessment (VISA; Porfeli et al., 2011). The Italian version of VISA (Aleni Sestito et al., 2015) consists of 30 items divided in three scales of 10 items according to Porfeli et al. (2011) vocational identity measure. The scales analyze the dimension of commitment, exploration and reconsideration of commitment: Each scale contains 2 subscales with 5 items: the dimension of career commitment has been distinguished in commitment making (i.e., “I have known for a long time what career is best for me”) and identification with commitment (i.e., “I chose a career that will allow me to remain true to my values”). The dimension of career exploration has been distinguished in exploration in breadth (i.e “thinking about how I could fit into many different careers”) and exploration in depth (i.e., “learning what I can do to improve my chances of getting into my chosen career”). Finally, the dimension of reconsideration, following the English version of VISA (Porfeli et al., 2011), has been divided in career commitment flexibility (i.e., “I will probably change my career goals”) and self-doubt (i.e., “I doubt I will find a career that suits me”). Participants responded to the items on 5-point Likert scale ranging from 1 (strongly disagree) to 5 (strongly agree). Cronbach’s alphas were 0.82 for Commitment Making, 0.84 for Identification with Commitment, 0.79 for Flexibility, 0.82 for Self-Doubt, 0.81 for Exploration in Breadth, and 0.75 for Exploration in Depth in the cited study.

### Data Analysis

For the validation of the Italian version of PHCII, we have run the following analyses:

– Structural analysis with using Confirmatory Factor Analysis
– Internal consistency analysis by calculating Cronbach’s alphas for PHCII subscales
– Invariance across gender with multi-group analysis
– Convergent and divergent validity of PHCI with VISA, calculating correlations between subscales.

## Results

To validate the Italian version of the PHCII, firstly we tested, using Confirmatory Factor Analysis (CFA), whether the five-factor structure of the PHCI, firstly identified by Kim et al. (2014) and then confirmed in the English version (Rhee et al., 2016; Lee et al., 2019), was also appropriate for the Italian version. Second, we examined reliabilities of the five subscales of the PHCII. Then, we tested whether this solution was consistent across genders. The last analysis we have run was meant to test the convergent and divergent validity of the PHCII with VISA. Here, we report descriptive statistics for PHCI 25 items in Table 1.

**Table 1:** Descriptive statistics for the 25 items of PHCI.

**Item**	**Mean**	**Std. Deviation**	**Skewness**	**Kurtosis**
PHCI1	3.621	0.824	−0.043	0.112
PHCI2	3.655	0.955	−0.542	0.131
PHCI3	3.792	1.057	−0.800	0.161
PHCI4	3.564	0.999	−0.331	−0.159
PHCI5	3.401	1.033	−0.353	−0.241
PHCI6	3.383	1.004	−0.611	0.105
PHCI7	3.360	1.081	−0.512	−0.399
PHCI8	3.404	1.122	−0.448	−0.547
PHCI9	3.933	0.858	−0.860	1.049
PHCI9	3.354	1.011	−0.408	−0.274
PHCI10	3.281	1.008	−0.424	−0.425
PHCI11	3.938	0.846	−0.577	0.156
PHCI12	3.939	0.806	−0.693	0.686
PHCI13	4.097	0.836	−0.837	0.644
PHCI14	4.076	0.852	−0.826	0.636
PHCI15	4.046	0.838	−1.081	1.870
PHCI16	4.053	0.829	−0.835	0.985
PHCI17	3.907	0.954	−0.812	0.582
PHCI18	3.716	1.034	−0.721	0.147
PHCI19	3.354	1.011	−0.409	−0.274
PHCI20	3.835	0.842	−0.578	0.539
PHCI21	3.851	0.876	−0.460	0.019
PHCI22	4.071	0.823	−0.999	1.695
PHCI23	3.916	0.919	−0.683	0.254
PHCI24	3.932	0.882	−0.699	0.613
PHCI25	3.998	0.872	−0.900	1.197

### Confirmatory Factor Analysis

Confirmatory factor analysis (CFA) was performed on the 25 items using the software jamovi (The Jamovi Project, 2021).^2^ Model fit was examined first with the overall sample. Various indices were used to evaluate model fit (Seffah et al., 2006): Goodness-of-Fit Index (GFI) should be equal or exceed 0.95 (Hu and Hu and Bentler, 1999), with values higher than 0.90 considered to be acceptable (Bentler, 1990); and the Root Mean Square Error of Approximation (RMSEA) should be equal or less than 0.08 (Browne and Cudeck, 1993).

#### Fit Indices for This Model Are Satisfactory

**Figure 1:**
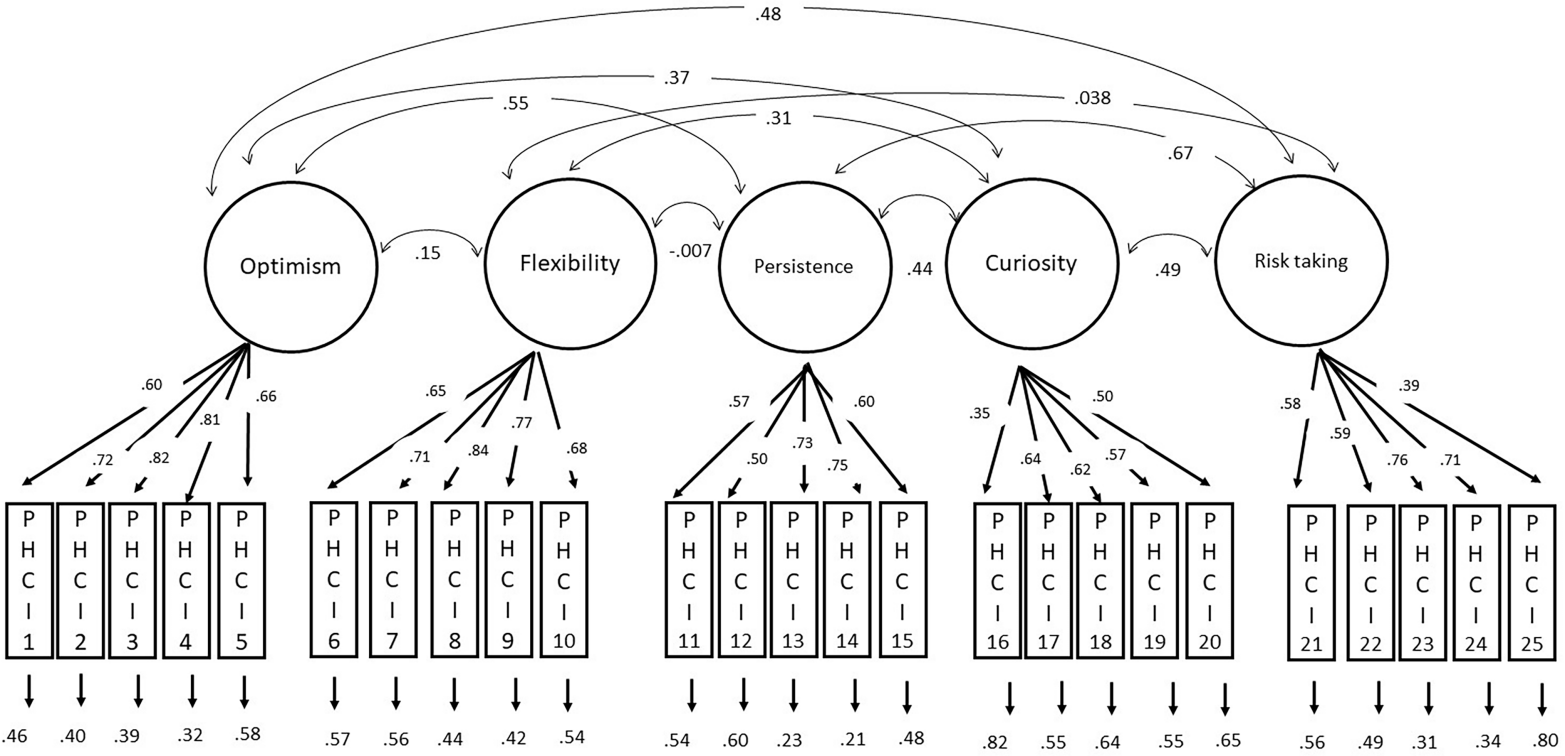
Standardized solution of the five-factor model of the PHCI.

Root mean square error of approximation (RMSEA) is equal to 0.067; Comparative Fit Index (CFI) is 0.982; Tucker-Lewis Index (TLI) is 0.980; Bentler-Bonett Normed Fit Index (NFI) is 0.976; Bollen’s Relative Fit Index (RFI) is 0.973; Bollen’s Incremental Fit Index (IFI) is 0.983; and Goodness-of-fit index (GFI) is 0.982. As a result, Figure 1 displays the five-factor standardized solution for the PHCI in the Italian sample. The covariances among the five subdimensions are reported in Table 2.

**Table 2:** Covariances: the first number refers to estimate, the one in parentheses to standard error and the third number is the *p* value.

	**Flexibility**	**Estimate**	**Std. Error**	**P**
		**Persistence**	**Curiosity**	**Risk-taking**
**Optimism**	0.151 (0.053) 0.004	0.455 (0.043) < 0.001	0.366 (0.051) < 0.001	0.480 (0.042) < 0.001
**Flexibility**		−0.007 (0.053) *0.895*	0.311 (0.054) < 0.001	0.038 (0.055) *0.488*
**Persistence**			0.439 (0.048) < 0.001	0.671 (0.032) < 0.001
**Curiosity**				0.489 (0.047) < 0.001

These results indicate a good fit of the five-factor model in the Italian sample. Then, we tested whether this factorial solution was consistent across genders.

### Reliability

The Italian version of the PHCII shows very good internal consistency evaluated with Cronbach’s indices. The five subscales have the following unidimensional reliability valued: Optimism *α* = 0.87, Flexibility *α* = 0.83, Persistence, *α* = 0.87, Curiosity *α* = 0.73, and Risk-Taking *α* = 0.82. The overall scale has *α* = 0.88.

These results indicate a good internal reliability for subscales in the Italian sample. The five-factor model of the 25 items of PHCI provided a good fit to the data and all items loaded significantly on their respective latent factors. The good Cronbach’s alphas confirmed the internal consistency of the instrument. The Italian version of the PHCII, therefore, proves to be valid for emerging adults in the Italian context.

### Testing for Invariance Across Genders

We explored invariance across gender imposing different constraints to the model, with the same number of factors for males and females. Posing equality constraint on loadings, we obtained the following indexes *χ*^2^ [503] = 1,369, *p* < 0.001, CFI = 0.980, TLI = 0.978, and RMSEA = 0.078 [0.073–0.084]. With the equality constraints on intercepts, we obtained the following indexes *χ*^2^ [479] = 1,323, *p* < 0.001, CFI = 0.980, TLI = 0.977, and RMSEA = 0.076 [0.071–0.082]. With the equality constraints on means, the model had these indexes: *χ*^2^ [484] = 1,323, *p* < 0.001, CFI = 0.980, TLI = 0.977, and RMSEA = 0.074 [0.068–0.079]. These data support adequately invariance implying equal role of factors for both male and females.

### Convergent and Divergent Validity

Findings (see Table 3) revealed that PHCII-Optimism is correlated with all VISA-Commitment dimensions, positively with Commitment Making and Identification and negatively with Commitment Flexibility and doubt. Flexibility is positively correlated with Explore Depth and Breadth and Commitment Flexibility, whereas it is negatively correlated with Commitment Making, Commitment Identification, and Commitment Doubt. Persistence is positively correlated with Commitment Making, Commitment Identification, and Explore Depth, and it has a negative correlation with Commitment Flexibility and Commitment Doubt. Curiosity is positively correlated with Commitment Identification, Explore Depth, and Explore Breadth. Risk-taking is positively correlated with Commitment Making, Commitment Identification, and Explore Depth, whereas it has a negative correlation with Commitment Flexibility and Commitment Doubt, as happens for Persistence.

**Table 3:** Correlation between PHCI and VISA dimensions.

	Optim	Flex	Persist	Curios	Risk-taking	Comm_making	Comm_id	Explore_depth	Explore_breadth	Comm_flex
Commitment making	0.311^**^	−0.290^**^	0.372^**^	0.039	0.360^**^					
Commitment identification	0.390^**^	−0.097^*^	0.394^**^	0.265^**^	0.418^**^	0.500^**^				
Explore in depth	0.043	0.108^*^	0.127^**^	0.236^**^	0.187^**^	0.143^**^	0.257^**^			
Explore in breadth	−0.006	0.385^**^	−0.035	0.248^**^	0.023	−0.205^**^	0.016	0.393^**^		
Commitment flexibility	−0.208^**^	0.455^**^	−0.247^**^	0.082	−0.131^**^	−0.418^**^	−0.315^**^	0.116^*^	0.387^**^	
Commitment doubt	−0.388^**^	−0.183^**^	−0.342^**^	−0.088	−0.290^**^	−0.358^**^	−0.432^**^	0.119	0.252^**^	440^**^

* Indicates a significativity at level *p* = 0.05;

** Indicates a significativity at level *p* = 0.01.

## Discussion

Starting from the general hypothesis according to which the career planned happenstance skills could be regarded as resources that do allow young people to cope with occupational concerns, the present study was articulated in two main goals. Regarding the first one, the cross-cultural validity of the Planned Happenstance Career for a sample of Italian late adolescents supports the consistency of PHCI factorial structure in the Italian sample, the results support the psychometric proprieties of Italian version of PHCII. The internal consistency scores of the Italian version of PHCI was very good with Cronbach’s indices subscales that ranged from 0.73 to 0.88. Also, the results about the Confirmatory factor analysis (CFA) indicate a good fit of the five-factor model in the Italian sample. Moreover, the multi-group invariance analyses on the final five-factor model shows that gender does not affect the factorial structure. These results are in line with the original study (Kim et al., 2014). The sample in the present study has adequate numerosity, but it is limited considering the number of courses attended. Regarding age, the scale is conceived for emerging adults, so we did not cover a wide age range for the present study, but it can be done in future research.

Considering the second goal, the validity of PHCII was also supported by the correlations between vocational identity status and the PHCII, which is consistent with the findings from Rhee et al. (2016) and Lee et al. (2019). In general, we can say that Visa dimensions have different relations with the five skills of Happenstance. Specifically, the present study showed positive correlations between PHCII-Optimism and VISA Commitment Making and Identification; Flexibility and VISA Explore Depth and Breadth and Commitment Flexibility; Persistence and VISA Commitment Making, Commitment Identification and Explore Depth; yet, between Curiosity and VISA Commitment Identification, Explore Depth and Explore Breadth; and Risk-taking and VISA Commitment Making, Commitment Identification and Explore Depth. These findings are in line with the relations reported by Rhee et al. (2016) confirming the hypothesis for which the exploration and commitment dimensions of vocational identity status were related to higher levels of persistence and optimism, whereas the reconsideration dimension was positively related to flexibility.

Even if this study extends previous studies on this topic and the results support the validity of the use of the HPCI also in the Italian context, some limitations must be considered. The instruments used were self-reported questionnaires, which may have led to biased answers given by an overestimated or underestimated participants’ state. Moreover, the sample was composed only by Italian late adolescents from a university in southern Italy. Finally, the cross-sectional design does not allow to study in deep the eventual changes in the associations between VISA dimensions and Skills of Happenstance, data that could come up from a longitudinal study (Rhee et al., 2016).

Despite these limitations, the findings confirming previous study and literature help to generalize the validity of the career planned happenstance skills (PHCI) to different populations. Moreover, findings seem to have practical implications in career guidance and counseling. Positive associations between VISA dimensions and skills of Planned Happenstance confirm that the latter one can be considered as an important resource for vocational identity commitment making process besides, in general, a resource to cope with unpredictable experiences.

In particular, the results of the present study indicate that Visa dimensions have different relations with the five skills of Happenstance. This reinforces the hypothesis that have been already proposed by Rhee et al. (2016) on the possibility of customizing career intervention. Starting from this, the intervention of career guidance and counseling could consider the visa identity status of the individual in order to work on the implementation of specific Happenstance skills associated with a specific Visa Status.

## Data Availability Statement

The raw data supporting the conclusions of this article will be made available by the authors, without undue reservation.

## Ethics Statement

The studies involving human participants were reviewed and approved by Cerp - Ethical Committee for Psychological Research at Department of Humanities “Federico II.” The patients/participants provided their written informed consent to participate in this study.

## Author Contributions

LS wrote the introduction of the article and revised critically the manuscript for important intellectual content. MP performed data analysis and wrote the data analysis and results of the article. TP wrote the discussion of the article and gave substantial contribution to acquisition of data. LS and MP contributed equally. All authors contributed to the article and approved the submitted version.

## Conflict of Interest

The authors declare that the research was conducted in the absence of any commercial or financial relationships that could be construed as a potential conflict of interest.

## Publisher’s Note

All claims expressed in this article are solely those of the authors and do not necessarily represent those of their affiliated organizations, or those of the publisher, the editors and the reviewers. Any product that may be evaluated in this article, or claim that may be made by its manufacturer, is not guaranteed or endorsed by the publisher.

## Footnotes

^1^Kim, B. (2012). Development and validation of the career-related planned happenstance scale (CPHS). Unpublished master’s thesis. Seoul, South Korea: Korea University.

^2^ https://www.jamovi.org

## Appendix 1

The Italian version of PHCI scale (PHCII).

1. La mia carriera futura sarà brillante.

2. Ho una visione positiva della mia futura carriera.

3. Penso che il futuro sia pieno di opportunità.

4. Ci saranno tante opportunità di carriera nel mio futuro.

5. Anche se la mia carriera non dovesse andare come ho progettato, sarà comunque un success.

6. Penso che, ad un certo punto, la mia carriera potrebbe anche cambiare.

7. Penso che i lavori possano cambiare in qualsiasi momento.

8. Sono flessibile nel considerare diverse opzioni piuttosto che perseguire solo un percorso di carriera.

9. Sono disponibile a considerare di cambiare il mio percorso lavorativo in funzione di quel che mi succede.

10. Tendo ad essere flessibile nel modo in cui prendo le decisioni di carriera.

11. Andrò per la mia strada con costanza, anche se dovessi incontrare difficoltà inaspettate mentre valuto i vari lavori possibili.

12. Mentre valuto quale carriera intraprendere, persisto nelle mie attività anche se incontro difficoltà.

13. Persisterei nei miei obiettivi nonostante ostacoli inattesi.

14. Porterei persistentemente avanti i miei obiettivi lavorativi, nonostante inaspettate difficoltà.

15. Provo con pazienza a conseguire i miei obiettivi di carriera, anche se affronto inaspettate difficoltà.

16. Mi interesso a ciò che accade intorno a me.

17. Mi intriga l’idea che brevi opportunità lavorative portino esperienze completamente nuove.

18. Generalmente mi incuriosiscono gli eventi inattesi.

19. Mi interessano quelle nuove attività che possono essere utili per scegliere e pianificare la mia carriera lavorativa.

20. Quando mi imbatto in nuove informazioni lavorative, le seguo con curiosità.

21. Proseguirò il percorso di carriera che ho scelto anche se i risultati non sono garantiti.

22. Sono pronto a correre dei rischi per raggiungere i miei obiettivi lavorativi.

23. Anche se non ci fosse alcuna garanzia di successo lavorativo, affronterei comunque le sfide.

24. Sono disponibile a correre rischi nonostante le conseguenze siano incerte.

25. Sono disponibile ad affrontare la sfida di provare nuove cose che potrebbero portarmi a migliori scelte di carriera.
